# Retracted: Synergistic Effect of Stereotactic Radiotherapy Combined with Karelizumab on Patients with Advanced NSCLC

**DOI:** 10.1155/2023/9759218

**Published:** 2023-01-25

**Authors:** Journal of Healthcare Engineering

*Journal of Healthcare Engineering* has retracted the article titled “Synergistic Effect of Stereotactic Radiotherapy Combined with Karelizumab on Patients with Advanced NSCLC” [1] due to concerns that the peer review process has been compromised.

Following an investigation conducted by the Hindawi Research Integrity team [2], significant concerns were identified with the peer reviewers assigned to this article; the investigation has concluded that the peer review process was compromised. We therefore can no longer trust the peer review process, and the article is being retracted with the agreement of the Chief Editor.

The authors do not agree to the retraction; the author Beibei Cheng could not be reached by the publisher using the e-mail address provided to the publisher with the article submission.
